# Platelet CD36 links overweight and a prothrombotic phenotype in patients with non-valvular atrial fibrillation

**DOI:** 10.3389/fcvm.2022.1066228

**Published:** 2022-11-18

**Authors:** Hua Wang, Wei-hong Yan, Lei Gong, Nian-peng Song, Chun-xiao Wang, Lin Zhong

**Affiliations:** ^1^Department of Cardiology, Yantai Yuhuangding Hospital, Qingdao Medical College, Qingdao University, Yantai, China; ^2^Laboratory of Cardiology, Yantai Yuhuangding Hospital, Qingdao Medical College, Qingdao University, Yantai, China

**Keywords:** platelet CD36, overweight, non-valvular atrial fibrillation, stroke, platelet activation

## Abstract

**Introduction:**

The pathophysiological mechanisms linking the overweight and prothrombotic state of non-valvular atrial fibrillation (NVAF) are incompletely understood. Our objective was to evaluate the effect of platelet CD36 on the risk of stroke associated with overweight in NVAF patients.

**Methods:**

A cross-sectional study enrolled 182 subjects with NVAF in two groups: normal weight (18.5 < body mass index(BMI) < 25.0 kg/m^2^) and overweight (BMI ≥ 25.0 kg/m^2^). Clinical data, medical history, vital signs, transthoracic echocardiography parameters, and medication were recorded. Biochemical characteristics including blood glucose and serum lipid were analyzed in the Laboratory.

**Results:**

The expression of platelet CD36 and integrin α_IIb_β_3_ was detected by flow cytometry. Among the 182 patients with NVAF, 68 (37.36%) were classified as normal weight, 114 (62.64%) as overweight. With an increase in BMI, waist-hip ratio, cholesterol, triglycerides, left atrium diameters, and the ratio of mitral inflow E velocity to myocardial e' velocity in the mitral annulus (E/e') increased significantly (*P* < 0.05). The mean fluorescent intensity of platelet CD36 increased significantly in overweight patients (*P* < 0.01), in line with platelet activation biomarkers (platelet integrin αIIbβ3). Platelet CD36 was positively correlated with BMI and platelet integrin αIIbβ3, respectively (*P* < 0.05). Additionally, platelet CD36 and BMI were independent risk factors for platelet activation in patients with NVAF.

**Conclusions:**

Platelet CD36 is speculated to mediate the complex crosstalk between overweight and platelet hyperactivity, leading to the prothrombotic state in overweight patients with NVAF. Platelet CD36 could be a potential target for preventing the prothrombotic state in overweight patients with NVAF.

## Introduction

Non-valvular atrial fibrillation (NVAF) is the most common cardiac arrhythmia, projected not only to have an impact on mortality and disability, but also to result in an increase in economic burden (1). Stroke is the most common underlying cause of death from NVAF. Overweight has emerged as an important modifiable independent risk factor for NVAF (2). For every 5-unit increase in body mass index (BMI), there was a 19 to 29% higher excess risk of incident AF, a 10% risk of postoperative AF, and a 13% risk of postablation AF (3). Furthermore, there was a trend for increased stroke rates at 3 and 6-year follow-up in patients with abdominal obesity (4). And obesity is important risk factors for the development of systemic thromboembolic events (5, 6). However, the pathophysiological mechanisms linking overweight and the prothrombotic state of NVAF are highly complex and remain incompletely understood.

It is well known that adipose tissue is not only a reservoir of energy, but also an active endocrine organ. It is a major source of neurohumoral activation that could promote oxidative stress, the release of pro-inflammatory mediators or adipokines. CD36 is a class B scavenger receptor that is involved in the pathogenesis of metabolic dysregulation in obesity, insulin resistance, and atherosclerosis. Genetic variation at the CD36 loci has been associated with obesity and lipid components of the metabolic syndrome (7). Platelet CD36, expressed at 20,000 copies per platelet that recognizes specific oxidized lipid motifs, was found to link hyperlipidemia, oxidant stress, and a prothrombotic phenotype. Podrez et al. suggested that platelet CD36 could serve as a sensor of specific oxidized phospholipids generated during oxidative stress, inducing an activating signal, and may result in platelet activation by subthreshold concentrations of physiological agonists (8). PCSK9 (proprotein convertase subtilisin/kexin 9) in plasma directly enhances platelet activation and *in vivo* thrombosis by binding to platelet CD36 and thus activating downstream signaling pathways (9). However, it remains unclear whether platelet CD36 contributes to the overweight-associated prothrombotic state in patients with NVAF.

Our previous reports showed that in the condition of oxidative damage, platelet CD36 could bind to microvesicles in a phosphatidylserine (PS)-dependent manner, thus triggering the MKK4/JNK2 signaling axis, activating platelets and amplifying oxidative stress (10). In the present study, we aimed to evaluate the effect of platelet CD36 on overweight-associated stroke risk or prothrombotic phenotype in NVAF patients.

## Methods

### Study population

A total of 182 patients diagnosed with NVAF were recruited consecutively from Qilu Hospital of Shandong University. Patients with rheumatic valvular heart disease, any history of malignancy, infectious disease or hormone replacement therapy were excluded from the study. The subjects were categorized according to baseline BMI: 68 subjects were classified as normal weight (18.5 < BMI < 25.0 kg/m^2^) and 114 (55.03%) as overweight (BMI ≥ 25.0 kg/m^2^). The study conformed with the Declaration of Helsinki and was approved by the institutional ethics committee. Written informed consent was obtained from all subjects and procedures were approved by the institutional ethics committees.

### Reagent

PE-cy™5-conjugated mouse anti-CD41a antibody (clone HIP8), fluorescein isothiocyanate (FITC)-conjugated PAC-1, PE-conjugated anti-CD36 antibody and isotype-matched control IgG were from BD Biosciences/Pharmingen (San Jose, CA, USA).

### Baseline characteristics

The clinical data, medical history, vital signs, parameters of transthoracic echocardiography and medication at admission were obtained from the patients' medical records. Venous blood samples were collected after overnight fasting. The biochemical characteristics including blood glucose and serum lipid were analyzed in the Laboratory.

### Flow cytometry of platelet integrin αIIbβ3 and CD36 expression

Expression of platelet integrin α_IIb_β_3_ and CD36 were detected by flow cytometry. The citrated whole blood (2.5 μL) was incubated with 5 μL PEcy5-conjugated anti-CD41a antibody and 5 μL FITC-conjugated PAC-1 antibody (for activated platelet integrin α_IIb_β_3_) in the dark for 15 min. For CD36 quantification, the platelet suspension was incubated with 5 μL PEcy5-conjugated anti-CD41a antibody and 5 μL PE-conjugated anti-CD36 antibody or isotype-matched control IgG.

### Statistical analysis

The Kolmogorov-Smirnov test was used to test for normal distribution. Continuous variables are expressed as means ± SD. Categorical variables are summarized as frequencies and percentages. Comparison between groups involved chi-square test (for categorical data) or independent samples *t*-test (for continuous data). The correlation between two variables was assessed by Pearson or Spearman correlation analysis. The effects of the different independent variables on platelet activation were assessed with the use of linear regression models. *P* < 0.05 was considered significant. Analysis involved SPSS v. 18.0 (SPSS, Chicago, IL).

## Results

### Patient characteristics in NVAF patients: Relationship to BMI

Among the 182 NVAF patients, 68 (37.36%) were classified as normal weight, 114 (62.64%) as overweight. The clinical and biochemical characteristics of the subjects classified by BMI are shown in Table 1. Significant differences were observed between groups, with increased BMI associated with higher levels of waist-to-hip ratio, cholesterol, and triglyceride (*P* < 0.05). As well, overweight patients are prone to have enlarged left atrium (LA) and increased E/e' (the ratio of mitral inflow E velocity to myocardial e' velocity in the mitral annulus) (*P* < 0.05). The medical history and application of antiplatelet agents, anticoagulant drugs, calcium channel blockers, beta-receptor blockers and statins had no significant differences between groups (*P* > 0.05) (Table 1).

**Table 1:** Baseline characteristics of NVAF patients by BMI Category.

	**Normal weight** **(*n* = 68)**	**Overweight** **(*n* = 114)**	***P* value**
Age in years	61.84 ± 11.05	59.92 ± 10.93	0.257
Male, n (%)	47 (69.11%)	71(62.28%)	0.423
SBP(mmHg)	132.87 ± 19.29	138.18 ± 19.08	0.077
DBP(mmHg)	80.24 ± 14.34	82.65 ± 13.13	0.265
BMI(kg/m^2^)	22.85 ± 2.06	28.19 ± 2.59	0.000
WHI	0.89 ± 0.05	0.93 ± 0.05	0.000
Glucose (mmol/L)	5.68 ± 2.20	5.86 ± 1.51	0.555
Cholesterol (mmol/L)	4.46 ± 1.03	4.83 ± 1.23	0.034
TG(mmol/L)	1.23 ± 0.54	1.78 ± 1.13	0.000
LDL-C(mmol/L)	2.60 ± 0.80	2.78 ± 0.91	0.161
Echocardiographic parameters
LAD (mm)	38.32 ± 5.20	41.38 ± 6.18	0.009
LVId (mm)	45.18 ± 6.18	46.07 ± 5.24	0.470
RAD(supra-inferior diameter)	49.50 ± 11.03	48.42 ± 7.06	0.624
RAD(left-right diameter)	42.27 ± 7.68	42.40 ± 6.59	0.935
RVId (mm)	25.45 ± 4.44	24.54 ± 4.12	0.316
IVST(mm)	11.76 ± 2.04	11.87 ± 1.48	0.788
LVPWT (mm)	10.62 ± 1.85	10.75 ± 1.45	0.711
LVEF(%)	58.28 ± 9.09	58.99 ± 8.00	0.693
E/e'	4.60 ± 1.55	6.03 ± 2.46	0.004
Medical history
CAD, *n* (%)	35 (51.47%)	53 (46.49%)	0.543
HT, *n* (%)	39 (57.35%)	82 (71.93%)	0.052
DM, *n* (%)	13 (19.12%)	36 (31.58%)	0.084
HF, *n* (%)	27 (39.71%)	32 (28.07%)	0.140
Stroke, *n* (%)	15 (22.06%)	21 (18.42%)	0.568
Medicine
Antiplatelet agents, *n* (%)	44 (64.71%)	78 (68.42%)	0.628
Anticoagulant agents, *n* (%)	23 (33.82%)	41 (35.96%)	0.873
Statin, *n* (%)	15 (22.06%)	32 (28.07%)	0.388
ACE inhibitors or ARBs, *n* (%)	36 (52.94%)	68 (59.65%)	0.439
Beta-receptor blockers, n (%)	47 (69.12%)	86 (75.44%)	0.390

Values are expressed as mean ± SD or number (%). Analyses are done by chi-square test (for categorical data) or independent samples *t*-test (for continuous data). SBP, systolic blood pressure; DBP, diastolic blood pressure; WHI, waist-to-hip ratio; BMI, body mass index; TG, triglyceride; LDL-C, low-density lipoprotein cholesterol; LAD, diameter of left atrium; LVId, internal diameter of left ventricle; RAD, diameter of right atrium; RVId, internal diameter of right ventricle; IVST, thickness of inter-ventricular septum; LVPWT, thickness of posterior wall of left ventricle; LVEF, left ventricle ejection fraction; E/e', the ratio of mitral inflow E velocity to myocardial e' velocity in the mitral annulus; CAD, coronary artery disease; HT, hypertension; DM, diabetes mellitus; HF, heart failure; ACE, angiotensin converting enzyme; ARB, angiotensin receptor blocker.

### Patients in the overweight group had significantly increased expression of platelet CD36 and platelet integrin αIIbβ3

**Figure 1:**
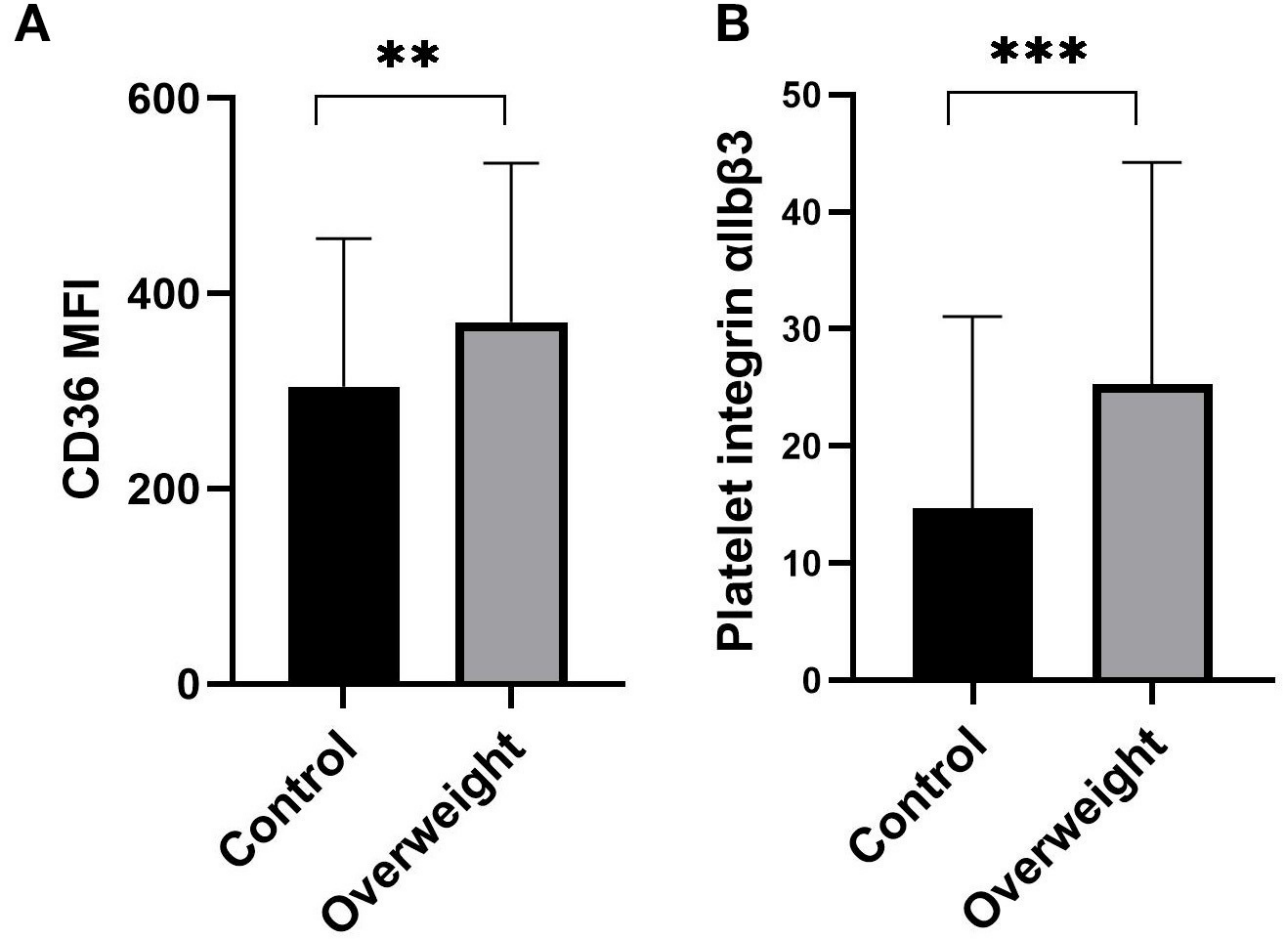
Expression of platelet CD36 and platelet integrin αIIbβ3. **(A)** Mean fluorescent intensity (MFI) of platelet CD36 increased significantly in overweight NVAF patients compared with that with normal weight (*P* < 0.01). **(B)** Platelet integrin αIIbβ3 enhanced with increased BMI (*P* < 0.01). Data are mean ± SD from at least 3 separate experiments. **P < 0.01; ***P < 0.001.

Mean fluorescent intensity (MFI) of platelet CD36 increased significantly in overweight patients (*P* < 0.01) (Figure 1A). Platelet activation in patients was assessed by surface detection of integrin αIIbβ3, which enhanced significantly in overweight group compared with those with normal weight (*P* < 0.001) (Figure 1B).

### The influence of platelet CD36 on platelet activation

**Figure 2:**
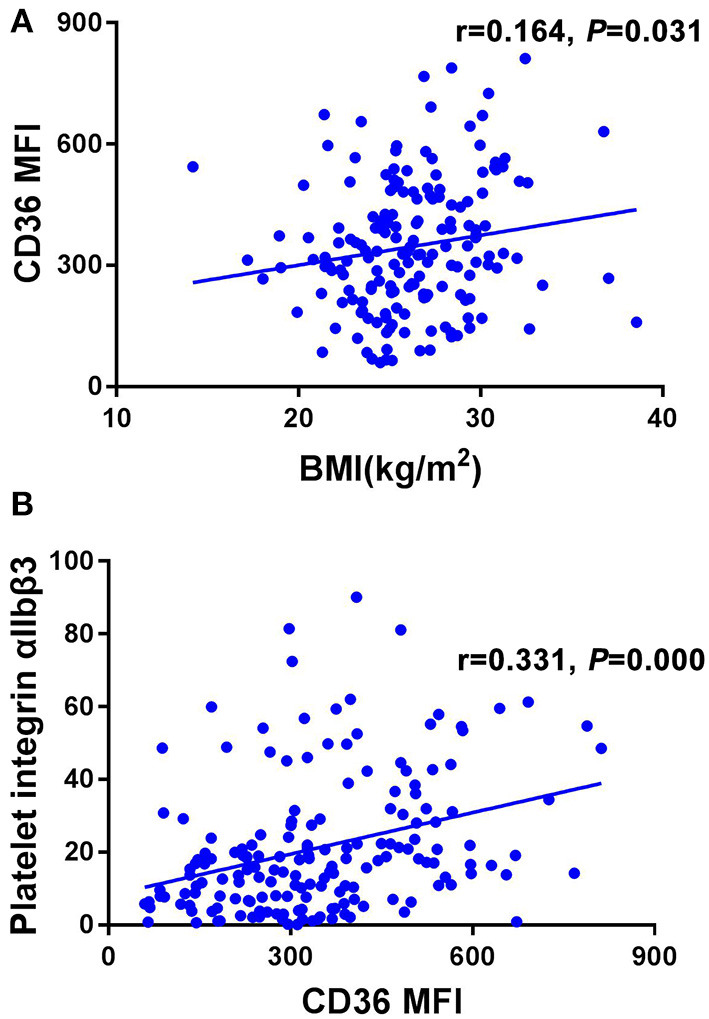
The relationship between BMI, platelet CD36 and platelet activation. **(A)** BMI was positive correlated with platelet CD36 significantly (r = 0.164, *P* = 0.031). **(B)** Platelet CD36 was positively correlated with platelet activation biomarkers (platelet integrin α_IIb_β_3_) (r = 0.331, *P* = 0.000).

Figure 2 shows the relationship between BMI, platelet CD36 and platelet activation. Pearson correlation analysis revealed a positive relationship of BMI with platelet CD36 (r = 0.164, *P* = 0.031) (Figure 2A); Platelet CD36 was positively correlated with platelet activation biomarkers (platelet integrin α_IIb_β_3_) (r = 0.331, *P* = 0.000) (Figure 2B).

### Multiple linear regression analysis of the correlation between the risk factors and platelet activation in NVAF patients

We introduced the following variables into the model for multiple linear regression analysis: platelet activation biomarkers (platelet integrin αIIbβ_3_) as the dependent variable; and age, BMI, systolic blood pressure (SBP), diastolic blood pressure (DBP), fasting glucose, total cholesterol, triglyceride (TG), low-density lipoprotein cholesterol (LDL-C) as the observed variables. The results indicated that platelet CD36 (β = 0.286, *P* = 0.001) and BMI (β = 0.175, *P* = 0.036) were independent risk factors for platelet activation in NVAF patients (Table 2).

**Table 2:** Multiple linear regression analysis for risk factors of platelet activation in NVAF patients.

**Variable**	**B**	**β**	**t**	***P***	**Adjusted R^2^**
Platelet CD36	0.032	0.286	3.472	0.001	0.114
BMI	0.862	0.175	2.123	0.036	

BMI, body mass index.

## Discussion

The present findings suggested that, compared to NVAF patients with normal weight, platelet CD36 MFI increased significantly in overweight patients. Platelet CD36 could serve as a mediator of overweight and prothrombotic state in NVAF patients, regardless of hypertension, diabetes, coronary artery disease, and heart failure. Furthermore, platelet CD36 could be one potential target to prevent prothrombotic state in obese patients with NVAF.

The rise in the prevalence of NVAF has coincided with the obesity epidemic. At present, the mechanisms of the risk of stroke associated with overweight in patients with NVAF remain incompletely understood. Obesity, a major component of metabolic disorders, has become one of the most important global health problems. In China, the overweight population increased by nearly 50% from 1992 to 2008 (11). It is well recognized that obesity in NVAF patients is associated with progressive inflammation, oxidative stress, and electrical and structural atrial remodeling promoted by the abnormal metabolic environment and the hemodynamic effects of increased BMI linearly associated with risk of stroke in NVAF (12). Height and weight were measured for the calculation of BMI for all patients. A BMI of 25 kg/m^2^ or higher was defined as overweight according to the criteria of the World Health Organization (WHO). The current study observed markedly higher levels of waist-hip ratio, total cholesterol, and triglyceride in patients with increased BMI suggesting the presence of dyslipidemia environment in overweight patients. Hyperlipidemia is associated with the process of platelet hyperactivity and increases the risk of prothrombotic state. PCSK9, a serine protease that plays a key role in lipid metabolism and increases plasma low-density lipoprotein cholesterol, binds to the CD36 receptor in platelets to enhance platelet activation by activating the p38/cPLA2/COX-1/TXA2 signaling pathways downstream of CD36 and increasing the generation of reactive oxygen species (ROS). ROS generation is characteristic of CD36 signaling in blood cells, including platelets (9, 13).

Furthermore, in line with other observations (14, 15), our work has shown that overweight patients with NVAF had a significantly enlarged LA and increased E/e', indicating that obesity promotes LA remodeling and diastolic dysfunction. Mahajan et al. have demonstrated that diastolic dysfunction and profibrotic environment lead to atrial fibrosis with sustained weight gain, whereas established interstitial fibrosis of LA may be reversible with weight reduction (16). It is postulated that infiltrated epicardial fat of the myocardium could promote obesity-related LA dilation and diastolic dysfunction. Furthermore, epicardial fat may have a paracrine effect on contiguous atrial tissue (17). CD36 is a fatty translocase acid protein that facilitates fatty acid uptake in various cell types, which is closely associated with obesity (18). In addition, CD36 may contribute to the pathogenesis of NVAF by activating monocyte-activated inflammation-associated signal pathways (19).

The mechanisms through which platelets become hyperactive and more prone to thrombus formation remain not fully understood in overweight patients with NVAF, which is of considerable importance. The procoagulant function of platelet CD36 has attracted attention in the past few years, being not only a scavenger receptor but also a signaling molecule. Enhanced platelet reactivity and risk of thrombosis may be associated with hyperlipidemia and enhanced oxidant stress in obese patients. Platelet CD36 potentiates thrombus formation under hyperlipidemic and oxidant stress conditions (20). CD36 ligands, such as ox-LDL, microparticles, and phosphatidylserine, can accumulate in plasma during hyperlipidemic conditions, where they can bind to platelet CD36 and thus modulate platelets tobecome hyperactive and more prone to thrombus formation (7). Several studies reached the same conclusions. Studies incubating normal platelets with plasma isolated from hyperlipidemic humans (which contain detectable oxidized lipids) show platelet activation in a CD36-dependent manner (13). Podrez et al. have shown that dyslipidemia enhances *in vivo* thrombosis, whereas genetic deletion of CD36 protects mice from hyperlipidemia-associated platelet activation and the accompanying prothrombotic phenotype (8). Studies by Gharib et al. demonstrated that CD36 deficiency prevented obesity-associated cardiac steatosis and insulin resistance, and reduced NADPH oxidase-dependent ROS production (21). In line with those studies, our previous work has demonstrated that the involvement of microvesicles with platelet CD36 triggers the MKK4/JNK2 signaling and contributes to platelet activation, while platelets deficient in CD36 could not be activated by microvesicles (22). In the current work, the platelet CD36 protein level was found to be higher in overweight NVAF patients than in normal weight patients regardless of hypertension, coronary artery disease, heart failure, and diabetes mellitus. The tendency to variation of platelet CD36 is consistent with that of platelet activation index (integrin αIIbβ3). BMI and platelet CD36, platelet CD36 and platelet integrin αIIbβ3 were positively correlated, respectively. To further explore the correlation between obesity and the prothrombotic state of NVAF, a multiple linear regression model was applied. Platelet activation as dependent variable, and age, blood pressure, BMI, WHI, fasting glucose, total cholesterol, triglyceride; low-density lipoprotein cholesterol and platelet CD36 independent variables, were introduced into the model for analysis. The results identified that platelet CD36 and BMI were risk factors for platelet activation in patients with NVAF. The finding of the present study indicated that platelet CD36 may be the critical mediator between obesity and the prothrombotic phenotype in patients with NVAF. In other words, platelet CD36 might be a potential target for preventing the prothrombotic state of overweight patients with NVAF.

One limitation of the present study is that prospective studies are needed to further evaluate the role of platelet CD36 in the inter-relationship between overweight and prothrombotic state of NVAF patients. Second, because the sample size was relatively small, the statistical power might have been low thus the results need to be further validated in large sample size studies.

## Conclusion

NVAF overweight and stroke risk are closely linked disorders. We found that platelet CD36 level was significantly increased in overweight patients with NVAF independent of well-known risk factors, such as hypertension, coronary artery disease, heart failure, and diabetes mellitus. Platelet CD36 is speculated to contribute to mediate complex crosstalk between adipose tissue and platelet hyperactivity, leading to a prothrombotic state in overweight patients with NVAF. Platelet CD36 could act as a new target to prevent the prothrombotic state of overweight patients with NVAF.

## Data availability statement

The original contributions presented in the study are included in the article/supplementary material, further inquiries can be directed to the corresponding author.

## Ethics statement

The studies involving human participants were reviewed and approved by the Ethics Committee of Qilu Hospital of Shandong University. The patients/participants provided their written informed consent to participate in this study.

## Author contributions

HW and N-pS designed the study, performed the experiments, and drafted the article. W-hY and LG analyzed the data and revised the article. C-xW and LZ collected the clinical data and performed the experiments. All authors finally contributed to the final approval of the version to be published, read, and approved the final manuscript.

## Funding

This work was supported by the research grants from the Science and Technology planning project of Yantai City (2021MSGY044, 2022MSGY075, and 2022MSGY067), the Shandong Provincial Medical and Health Plan (202103050906), the National Natural Science Foundation of China (82200503, 81900310, and 81701246), and the China International Medical Foundation (2021-N-15-38).

## Conflict of interest

The authors declare that the research was conducted in the absence of any commercial or financial relationships that could be construed as a potential conflict of interest.

## Publisher's note

All claims expressed in this article are solely those of the authors and do not necessarily represent those of their affiliated organizations, or those of the publisher, the editors and the reviewers. Any product that may be evaluated in this article, or claim that may be made by its manufacturer, is not guaranteed or endorsed by the publisher.
